# Supplementary material for: Chromothripsis during telomere crisis is independent of NHEJ, and consistent with a replicative origin
Source: Genome Res. 2019 May;29(5):737–49. doi: 10.1101/gr.240705.118 (PMC6499312; doi:10.1101/gr.240705.118)
Supplement: Supplemental Material [file supp_gr.240705.118_Supplemental_file_1.zip › contigs/annotated_contigs/DB114/contig.2.DB114_length_547_mean_cov_5.7550274223.docx]

**DB114_length_547_mean_cov_5.7550274223**

```
TAGCCAGGCGTGGTGGTGTGTGCCTGTAATCCCAGCTAATCAGGAGGCTGAGGCAGGAGAATAGCTTGAACCCTAGCTAGGAGGCGGAG
 >chr8:49019884-49020117 - E=1e-128 p=1e-03
GTTGCAGTGAGCCAAGATCGCACCATTGCACTCGAGCTGGGGCAACAAAGCAAGATTCTGTCTCAAAAAAAGAAAAGAAGAAGAAAACA

AAAAGCCTGTGTACATCAAGGTAAAAAGTTCAAGTCACCCATAAGTAGAGGCAGC|CCCAGAGAATCA|TGCCCCAGAGAATCATGGGC
                                                                    >chr8:49104520-4910
TGTTCTCCAAGAAACTGGAAACTAGAGTGCTCACTCCCAGGCCTGAAGCACACAGTTTTCTCATGAATGCCTCTCTGTTGTGTTTTGTA
4822 - E=4e-170
TGATTTTGCCGCATGACATTTGTAGTAATTCAGGCCTGTTCAAGTTGAATTCTTTGCAAGTTGCCTTATTCTCCCTTTCCTGAGATTTT

GTACATATTGTTTTCCTGAGTAATGGTATTTCTAAGTTAATTTCTTCTAATCAGAGCTACCAAAATGTACGTGCTTTCAGTAAACTCCT

ATTTGGTTTTGTTTT
```
